# 3D Collagen Fiber Concentration Regulates Treg Cell Infiltration in Triple Negative Breast Cancer

**DOI:** 10.3389/fimmu.2022.904418

**Published:** 2022-06-14

**Authors:** Huan Gao, Qi Tian, Yan Zhou, Lizhe Zhu, Yinliang Lu, Yingying Ma, Jinteng Feng, Yina Jiang, Bo Wang

**Affiliations:** ^1^ Department of Medical Oncology, The First Affiliated Hospital of Xi’an Jiaotong University, Xi’an, China; ^2^ Department of Radiology, The First Affiliated Hospital of Xi’an Jiaotong University, Xi’an, China; ^3^ Department of Breast Surgery, The First Affiliated Hospital of Xi’an Jiaotong University, Xi’an, China; ^4^ Department of Radiation Oncology, The First Affiliated Hospital of Xi’an Jiaotong University, Xi’an, China; ^5^ Department of Pathology, The First Affiliated Hospital of Xi’an Jiaotong University, Xi’an, China; ^6^ Center for Translational Medicine, The First Affiliated Hospital of Xi’an Jiaotong University, Xi’an, China

**Keywords:** triple negative breast cancer, tumor microenvironment, regulatory T cells, extracellular matrix, tumor-infiltrating lymphocytes, prognostic model

## Abstract

**Background:**

Triple negative breast cancer (TNBC) is characterized by poor prognosis and a lack of effective therapeutic agents owing to the absence of biomarkers. A high abundance of tumor-infiltrating regulatory T cells (Tregs) was associated with worse prognosis in malignant disease. Exploring the association between Treg cell infiltration and TNBC will provide new insights for understanding TNBC immunosuppression and may pave the way for developing novel immune-based treatments.

**Materials and Methods:**

Patients from TCGA were divided into Treg-high (Treg-H) and Treg-low (Treg-L) groups based on the abundance of Tregs according to CIBERSORT analysis. The association between expression level of Tregs and the clinical characteristics as well as prognosis of breast cancer were evaluated. Next, a Treg-related prognostic model was established after survival-dependent univariate Cox and LASSO regression analysis, companied with an external GEO cohort validation. Then, GO, KEGG and GSEA analyses were performed between the Treg-H and Treg-L groups. Masson and Sirius red/Fast Green staining were applied for ECM characterization. Accordingly, Jurkat T cells were encapsulated in 3D collagen to mimic the ECM microenvironment, and the expression levels of CD4, FOXP3 and CD25 were quantified according to immunofluorescence staining.

**Results:**

The expression level of Tregs is significantly associated with the clinical characteristics of breast cancer patients, and a high level of Treg cell expression indicates a poor prognosis in TNBC. To further evaluate this, a Treg-related prognostic model was established that accurately predicted outcomes in both TCGA training and GEO validation cohorts of TNBC patients. Subsequently, ECM-associated signaling pathways were identified between the Treg-H and Treg-L groups, indicating the role of ECM in Treg infiltration. Since we found increasing collagen concentrations in TNBC patients with distant migration, we encapsulated Jurkat T cells within a 3D matrix with different collagen concentrations and observed that increasing collagen concentrations promoted the expression of Treg biomarkers, supporting the regulatory role of ECM in Treg infiltration.

**Conclusion:**

Our results support the association between Treg expression and breast cancer progression as well as prognosis in the TNBC subtype. Moreover, increasing collagen density may promote Treg infiltration, and thus induce an immunosuppressed TME.

## Introduction

Breast cancer ranks as the leading cause of cancer-associated death among women worldwide (1). Thanks to the progress of targeted therapy and immunotherapy, the past few decades have witnessed great improvement in the treatment of breast cancer. However, metastasis is still exceedingly difficult to inhibit owing to the high heterogeneity of breast cancer. According to the histopathological expression level of hormone receptors and Her2 gene amplification, breast cancer has been classified into three main molecular subtypes: luminal, Her2 enrichment, and triple-negative breast cancer (TNBC) (2), among which TNBC is characterized by poor prognosis and lack of effective therapeutic agents owing to the absence of target biomarkers (3). These difficulties highlight the critical need for exploring more efficient biomarkers in TNBC.

Recently, efficient treatments are still limited due to the high heterogeneity in TNBC (4). Significant efforts have been devoted to establish the prognostic model based on tumor-infiltrating lymphocytes (TILs) and immune-related gene expression signatures (5–7). According to the transcriptomic analysis of 386 original TNBC patients, combined with external METABRIC validation cohorts, TNBC was classified into 3 distinct immune clusters: the “immune-desert” cluster, the “innate immune-inactivated” cluster and the “immune-inflamed” cluster (7). These results reveal the high heterogeneity of TNBC immune microenvironment, which indicates an association between TNBC progression and immunosuppression.

Regulatory T cells (Tregs), a specialized subset of T cells that express CD4, CD25 and forkhead box protein P3 (FOXP3), act as key mediators of immunologic tolerance. In particular, Tregs obtain an immunosuppressive capacity after exposure to inflammatory conditions in the tumor microenvironment (TME), which then suppress autoimmunity by inhibiting T cell proliferation and cytokine production (8–10). Additionally, the depletion of Tregs could inhibit tumor growth (11, 12), suggesting the promoting role of Tregs in TNBC progression. Thus, it is of critical importance to establish Treg-related signatures for identifying patients who are resistant to immunotherapy.

As a key component in the TME, ECM provides structural support for resident cells and dynamically regulates the bioprocess of breast cancer cells (13, 14). Evidently, increasing ECM stiffness promotes the invasion phenotypes of mammary epithelial cells *via* an integrin-Rho-YAP/TAZ-dependent pathway (13, 15). Collagen type I consists of the main component of the tumor ECM that provides the main skeleton for the TME and has been reported to promote TNBC metastasis (16). Mechanistically, the binding of DDR1-ECD to collagen enforces aligned collagen fiber organization and obstructs the immune response (17). In addition, high collagen density can significantly inhibit T-cell proliferation and lead to an increased ratio of CD4+ to CD8+ T cells (18). The above evidence reveals that the ECM may play a critical role in immunosuppression, which leads us to wonder the association between the ECM and Tregs. Thus, new work focusing on this issue holds great promise for exploring potential therapeutics.

In the present study, based on a comprehensive analysis of TCGA breast cancer patients, our initial results indicate the prognostic value of Tregs in TNBC patients. To further demonstrate this, a prognostic Treg-related risk score model was established using the independent cohort. The new signature classification accurately predicted outcome both in TCGA training and GEO validation cohorts of TNBC patients. Subsequently, based on the RNA-seq analysis between different Treg groups, ECM-associated signaling pathways were identified, indicating the role of ECM in Treg cell infiltration. Based on the increased collagen concentration according to our clinical tissue validation, we encapsulated Jurkat T cells within a 3D matrix with different collagen concentrations and found that increasing collagen concentrations promoted the expression of Treg biomarkers, supporting the regulatory role of the ECM in Treg cell infiltration. Importantly, our findings may provide new insights into the mechanism of immunosuppression.

## Materials and Methods

### Collection of Data Sources and Grouping of Breast Cancer Patients

The RNA sequence expression profile and corresponding clinical annotations of 1109 breast cancer samples and 113 paracancerous samples were obtained from the TCGA database (https://portal.gdc.cancer.gov). The microenvironmental status of the paracancerous tissues and breast cancer tissues was analyzed on TIMER2.0 (http://timer.cistrome.org/). The CIBERSORT algorithm (19), a method for characterizing the cell composition of complex tissues based on gene expression profiles, was selected for quantification of the absolute abundance of B cells, T cells, natural killer cells, macrophages, dendritic cells, eosinophils, and neutrophils. Then, the patients were classified into Treg-H and Treg-L groups according to the median Treg expression. To validate the prognostic value of the potential signature obtained from the TCGA database according to Treg expression, gene expression profiles of patient-derived triple-negative breast cancer tissues (GSE58812, GPL570, n = 107) from the GEO database were also selected.

### Evaluation of the Association Between Treg Expression and Clinical Characteristics

According to the RNA sequence expression and clinical data of breast cancer samples in the TCGA database, the expression level of Tregs between paired/unpaired adjacent normal and cancer tissues were compared. Moreover, the associations between Treg expression and clinical characteristics, including T, N, M and AJCC anatomic stages, were evaluated.

### Initial Verification of the Prognostic Value of Treg Expression

To verify the prognostic value of Treg expression, the R software “survival” package was used to compare the overall survival (OS) and disease-free survival (DFS) of breast cancer patients in the Treg-H and Treg-L groups. Kaplan–Meier curves were plotted to visualize the OS and DFS between each group in all breast cancer patients as well as the luminal and TNBC subtypes, and the log-rank test was performed to evaluate whether the differences in survival time were significant according to the corresponding *p* value.

### Identification of Differentially Expressed Genes (DEGs) and Screening of Prognostic DEGs

To identify the DEGs between different Treg groups in TNBC patients, the “limma” package calculation in R software was performed, and genes with cutoff screening of |log2FC| > 0.6 and *p* < 0.05 according to the Wilcoxon test were termed DEGs. Since the DEGs were identified based on Treg expression, we further compared the expression level of an immune-related gene set (**Table S1**) downloaded from the IMMUPORT database (https://www.immport.org/home) and biomarkers of Tregs between different Treg groups. Coexpression analysis between DEGs and immune-related transcription factors (TFs) (**Table S1**) downloaded from the Cistrome database (http://www.cistrome.org/) was performed. Then, the potential prognostic DEGs were identified using univariate Cox regression analysis with the survival R package.

### Establishment and Validation of the Treg-Related Prognostic Model

The significant survival-related genes were subsequently included in the least absolute shrinkage and selection operator (LASSO) estimation to determine the core prognostic genes by the glmnet package with 1000 repetitions for construction of the survival modeling. The specific risk score for each TNBC patient was calculated following the formula:


$$n_{1}*ExpGene_{1}+\;n_{2}*ExpGene_{2}+\;\ldots \;\ldots \;+n_{x}*ExpGene_{x}$$

where *n* is the regression coefficient of core prognostic genes obtained by LASSO regression, and *ExpGene* represents the expression level of specific core genes. Patients were classified into high- and low-risk groups according to the median value of the risk score. Next, the formula was applied to an external GEO cohort of TNBC patients to verify the validity and reproducibility of the constructed risk model. Kaplan–Meier curves were plotted to visualize the OS between the high- and low-risk groups in the TCGA training and GEO validation cohorts. Time-dependent receiver operation characteristic (ROC) curves were generated to assess the accuracy of the prognostic model based on Treg expression. Combining the risk scores with other clinical characteristics, a nomogram was constructed using the R rms package to calculate the OS of 1-, 3-, and 5-year-old TNBC patients. Calibration curves were generated to evaluate the reliability of the established nomogram.

### Functional Pathway Analysis Based on Treg Expression in TNBC

Then, to reveal the potential pathway between the Treg-H and Treg-L groups, gene ontology (GO) terms, including biological processes (BPs), cellular components (CCs), and molecular functions (MFs), were analyzed by the R “clusterProfiler” package to determine the biological functions of DEGs. To explore the possible enriched signaling pathways of DEGs, KEGG enrichment analysis was carried out utilizing the “clusterProfiler” package of R software with a statistical threshold of *p* < 0.05. In addition, Reactome pathway analysis was performed by Gene Set Enrichment Analysis (GSEA 4.1.0).

### ECM Characterization of Breast Cancer Tissues

Based on the identified signaling pathways, we assessed the specific ECM characteristics by Masson’s trichrome staining (Solarbio, China) and Sirius red/Fast Green staining (Solarbio, China) to identify the corresponding ECM characteristics of breast cancer tissues with distant metastasis. ECM collagen fibers were red-stained by Sirius red/fast green staining and blue-stained by Masson’s trichrome. The percentage of collagen area was estimated by ImageJ, and the organization of the collagen fibers was evaluated by fiber orientation distribution using the ImageJ plugin Orientation J.

### 3D Culture in Different Collagen Concentration Gels

After conforming to the guidelines set by the Institutional Animal Care Committee of Xi’an Jiaotong University, collagen fibers were harvested from rat tail tendons as described previously. The human breast cancer cell line MDA-MB-231 was obtained from American Type Culture Collection (ATCC, Manassas, VA) and cultured in L-15 supplemented with fetal bovine serum (FBS; Gibco, Grand Island, NY, USA), penicillin, and streptomycin (Sigma, St. Louis, MO, USA). Jurkat T cells were purchased from the Cell Bank of the Chinese Academy of Sciences (Shanghai, China). The culture medium of Jurkat T cells was RPMI 1640 (HyClone, Logan, UT, USA) supplemented with FBS, penicillin, and streptomycin. Both cell types were used between passages 2–20. Jurkat T cells were capsulated with different collagen concentrations (1 mg/ml vs. 3 mg/ml vs. 6 mg/ml). For cell activation conditions, the supernatant of MB-231 cells was added to Jurkat T cells for 24 h. All cells were maintained at 37°C in a humidified 5% CO_2_ atmosphere.

### Immunofluorescence Staining and Confocal Microscopy

Cells were encapsulated within a 3D collagen matrix with different collagen concentrations. The cells were fixed in 4% formaldehyde, permeabilized with 0.5% Triton X-100 for 10 min, blocked in 1% BSA for 1 hour, and then incubated with a 1:1000 dilution of CD4 antibody (#48705, Cell Signaling Technology, 4°C, 2 h), a 1:100 dilution of Foxp3 monoclonal antibody (ab275120, Abcam, 4°C, 2h). For CD25 detection, 3D samples were incubated with CD25 antibody (#39475, Cell Signaling Technology, 4°C, overnight), followed by secondary antibody (#3978, Cell Signaling Technology, RT, 2h), and then incubated with 4’,6-diamidino-2-phenylindole (DAPI, Invitrogen) to stain cell nuclei for 15 min. Confocal fluorescence microscopy was performed using an Olympus FV300. The fluorophores were excited by 405, 488 and 561 nm laser lines.

### Statistical Analysis

All RNA sequence data were analyzed using R version 4.1.1. Quantification data of collagen fibers were analyzed by ImageJ and plotted by GraphPad Prism 8, while the orientation distribution of collagen fibers was plotted by Origin 9.1. All cell culture experiments were performed at least three times independently. Student’s two-sided t test was performed to compare the differences between groups, and the results are presented as the mean ± standard deviation (SD). Differences in survival between different risk groups were compared by Kaplan–Meier curves followed by the log-rank test. *p* < 0.05 was considered statistically significant.

## Results

### Treg Expression Is Associated With the Clinical Characteristics of Breast Cancer Patients

**Figure 1:**
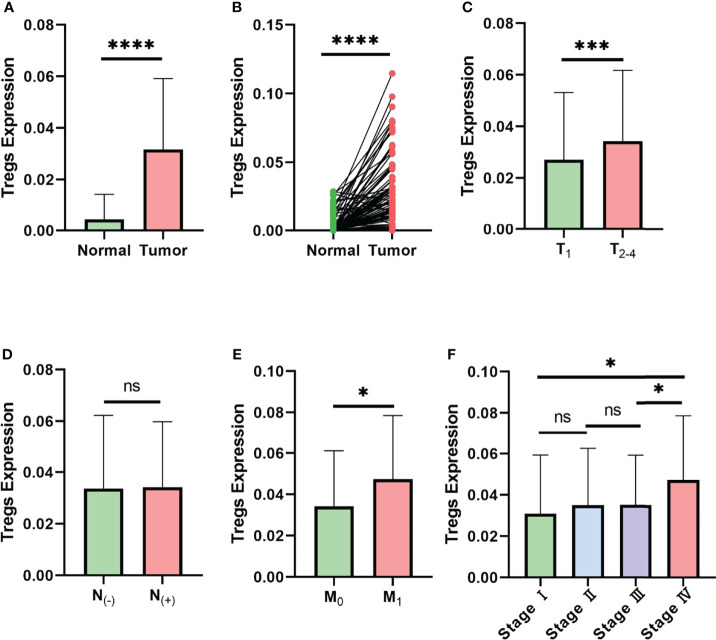
Expression level of Tregs is associated with the clinical characteristics of breast cancer in TCGA database. **(A)** Expression level of Tregs between normal and cancer tissues of breast cancer patients in TCGA database. **(B)** Expression level of Tregs between paired normal and cancer tissues of breast cancer patients in TCGA database. **(C)** Expression level of Tregs in different T stages of breast cancer patients. **(D)** Expression level of Tregs among nodal status-negative and nodal status-positive breast cancer patients. **(E)** Expression level of Tregs in breast cancer with or without distant metastasis. **(F)** Expression level of Tregs between different AJCC anatomic stages. ns, not significant; *p < 0.05, ***p < 0.001, ****p < 0.0001.

To predict whether Treg expression is associated with tumorigenesis and clinical characteristics of breast cancer, CIBERSORT analysis was performed in 980 breast cancer samples and 113 paracancerous samples from TCGA (**Figure S1**). Compared with the paracancerous samples, cancer samples displayed higher levels of Treg expression in both paired and unpaired breast cancer tissues (**Figures 1A, B**). The results also indicate that a higher expression level of Tregs is associated with higher T stage (**Figure 1C**) and M stage (**Figure 1E**), while similar levels remain in nodal status-negative and positive patients (**Figure 1D**). Consistent with the variation in M_0_ and M_1_ patients, patients in AJCC anatomic stage IV had a higher expression level of Tregs in general (**Figure 1F**). Taken together, these results indicate an association between Treg expression and the clinical characteristics of breast cancer patients.

### A High Level of Treg Expression Predicts a Worse Prognosis in TNBC Patients

**Figure 2:**
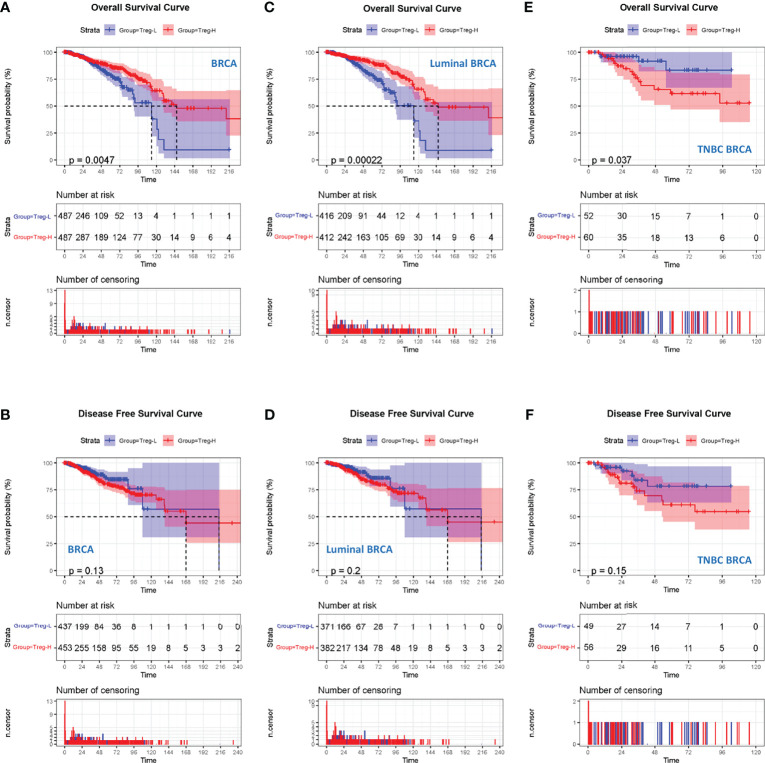
High Treg expression is associated with a worse prognosis in TNBC. **(A)** Kaplan–Meier curves for the OS of breast cancer patients in TCGA database who were divided into Treg-H and Treg-L groups. **(B)** Kaplan–Meier curves for the DFS of breast cancer patients between the Treg-H and Treg-L groups. **(C)** Kaplan–Meier curves for the OS of the luminal subtype between the Treg-H and Treg-L groups. **(D)** Kaplan–Meier curves for the DFS of the luminal subtype between the Treg-H and Treg-L groups. **(E)** Kaplan–Meier curves for the OS of the TNBC subtype between the Treg-H and Treg-L groups. **(F)** Kaplan–Meier curves for the DFS of the TNBC subtype between the Treg-H and Treg-L groups.

Since the above data suggest an association between Treg expression and the clinical stages of breast cancer patients, we next wondered whether Treg expression contributes to the prognosis of breast cancer. We classified the 980 breast cancer samples into 490 Treg-H and 490 Treg-L patients based on the median Treg expression level. The Kaplan–Meier curve showed that the patients in the Treg-H group exhibited better overall survival (OS) than those in the Treg-L group, while the disease-free survival (DFS) was similar in both groups (**Figures 2A, B**).

As the results are in contrast with the high level Treg expression in the cancer tissues, we wondered whether Tregs were only associated with a specific molecular subtype of breast cancer patients. Thus, we evaluated the prognostic value of Treg expression in luminal and TNBC molecular subtypes (**Figures 2C–F**). Interestingly, the OS and DFS results in luminal breast cancer patients were consistent with those in total breast cancer patients (**Figures 2C, D**). However, in TNBC patients, the Treg-H group showed a significantly worse OS (*p* = 0.037) than the Treg-L group. Consistently, the DFS of Treg-H patients also showed a worse trend, although the p value was not statistically significant (*p* = 0.15). As reported previously, Tres heavily infiltrate TNBC, and their accumulation is affected by metabolic reprogramming in TNBC cancer cells (20). Together with our OS and DFS results, this evidence suggests the prognostic value of Treg expression in TNBC.

### Establishment of a Prognostic Model Based on Treg Expression

**Figure 3:**
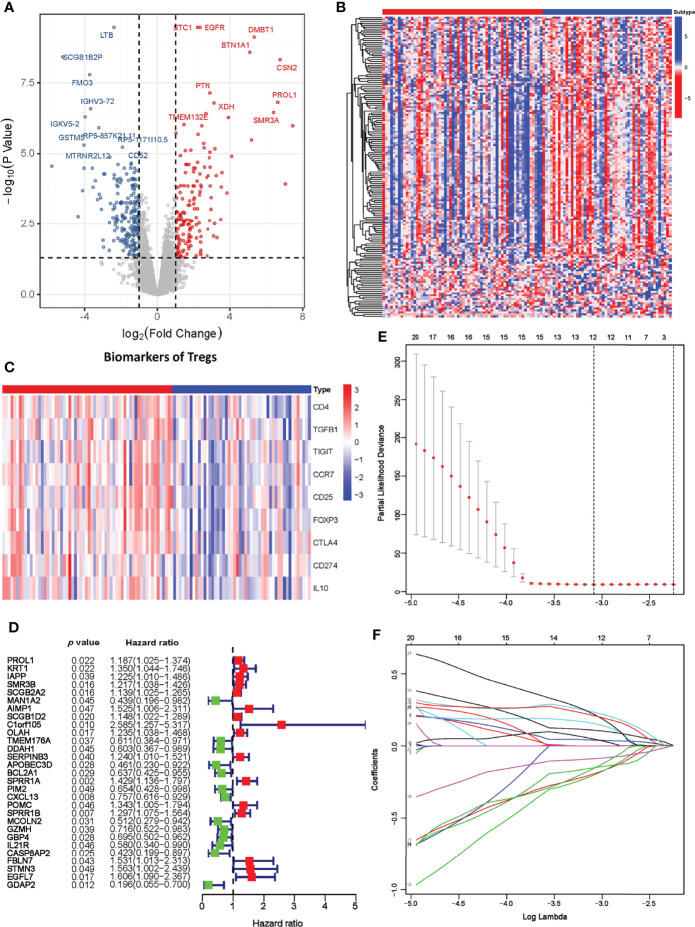
Establishment of the Treg-related prognostic model. **(A)** Volcano plot of differentially expressed genes across the Treg-H and Treg-L groups. **(B)** Heatmap of immune-related gene expression in the Treg-H and Treg-L groups. **(C)** Heatmap of the expression levels of Treg biomarkers in the Treg-H and Treg-L groups. **(D)** Prognostic-associated DEGs identified by univariate Cox regression. **(E, F)** The prognostic signature constructed by LASSO regression.

As our results suggest a critical role of Treg expression in TNBC progression and prognosis, we next constructed a prognostic model based on Treg expression. A total of 793 differentially expressed genes, including 245 upregulated and 383 downregulated genes, were identified, and the volcano plot displays those DEGs with FDR < 0.05 and |log2FC| > 1 (**Figure 3A**). We also evaluated the expression level of an immune gene set and biomarkers of Tregs in the two groups, and the heatmaps consistently showed broad variation (**Figures 3B, C**).

We next performed univariate Cox regression analysis on the DEGs and extracted the prognosis-associated DEGs (**Figure 3D**). The prognostic DEGs were then used for the following LASSO Cox regression analysis. Based on the optimal penalty parameter (λ) shown in **Figures 3E, F**, the Treg-related prognostic model based on 12 core prognostic genes was established following the formula: 0.040 * Exp ***_SCGB2A2_*** + 0.154 * Exp ***_AIMP1_*** +0.108*Exp ***_OLAH_*** + (-0.193) * Exp ***_TMEM176A_*** + (-0.202) * Exp ***_DDAH1_*** + 0.234 * Exp ***_SPRR1A_*** + (-0.089) * Exp ***_CXCL13_*** + 0.047 * Exp ***_POMC_*** + (-0.156) * Exp ***_MCOLN2_*** + 0.219 * Exp ***_STMN3_*** + 0.209 * Exp ***_EGFL7_*** + (-0.152) * Exp ***_GDAP2_***. Subsequently, TNBC patients were classified into a high-risk group (*n* = 56) and a low-risk group (*n* = 56) on the basis of their median risk score.

### An External GEO Dataset Validation and Nomogram Model Support the Prognostic Value of the Risk Score

**Figure 4:**
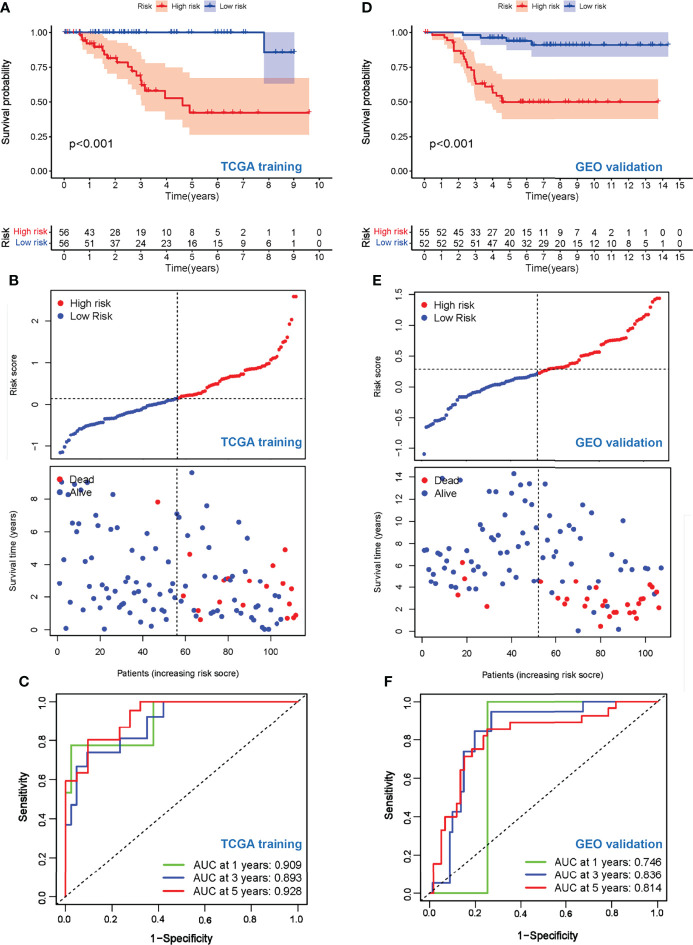
Evaluation of prognostic values of the risk scores. **(A)** Kaplan–Meier curve of OS for patients in the Treg-related high-risk and low-risk subgroups in the TCGA training cohort. **(B)** The distributions of risk scores and OS status in the TCGA training cohort. **(C)** Time-dependent ROC curve of the prognostic values of the TCGA training cohort in 1-, 3-, and 5-year OS. **(D)** Kaplan–Meier curve of OS for patients in the Treg-related high-risk and low-risk subgroups in the GEO validation cohort. **(E)** The distributions of risk scores and OS status in the GEO validation cohort. **(F)** Time-dependent ROC curve of the prognostic values of the GEO validation cohort in 1-, 3-, and 5-year OS.

To test the prognostic value of our risk model, the OS of the high- and low-risk groups was compared, and the results showed a significantly better OS in the low-risk group (*p* < 0.001) (**Figure 4A**). The living condition of TNBC patients and their risk score distribution further support the better OS of the low-risk group (**Figure 4B**). The AUC for the signature of OS at 1 year was 0.909, and it was 0.893 at 3 years and 0.928 at 5 years (**Figure 4C**).

**Figure 5:**
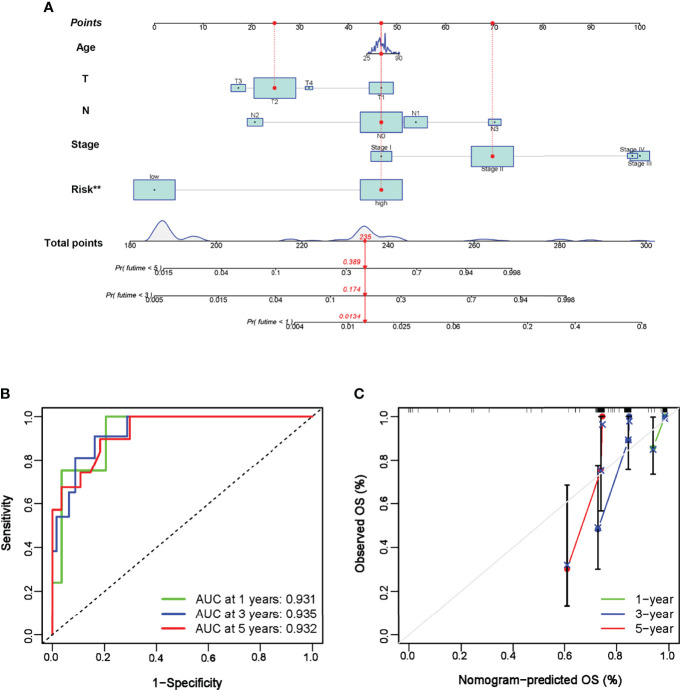
Establishment of nomogram prediction. **(A)** A nomogram was constructed to calculate the OS at 1, 3, and 5 years in TNBC patients. **(B)** Time-dependent ROC curve of the nomogram in 1-, 3-, and 5-year OS. **(C)** Calibration curves of the established nomogram. **P < 0.01.

To further explore the stability and reproducibility of the prognostic value, the risk score model was applied to the GEO validation cohort with 107 TNBC patients. According to the median-risk score obtained in TCGA, patients in the validation GEO cohort were split into high-risk (*n* = 55) and low-risk (*n* = 52) groups. There was a significant statistical distinction in the Kaplan–Meier survival curves of the two groups, which consistently supported a better prognosis of the low-risk group (**Figure 4D**). The distribution of risk scores and survival status of the GEO validation cohort were in accordance with those of the TCGA training set (**Figure 4E**). In the entire validation set, the AUC was 0.746 at 1 year, 0.836 at 3 years, and 0.814 at 5 years (**Figure 4F**). Collectively, these results suggest that our prediction model could achieve superior fitting effects both in the TCGA training and GEO validation datasets. Afterwards, clinical characteristics and risk scores were combined into the nomogram, which showed that the risk score was the most influential factor among the nomograms’ total scores (**Figures 5A–C**). The AUC for the nomogram signature of OS at 1 year was 0.931, and it was 0.935 at 3 years and 0.932 at 5 years (**Figure 5B**).

### ECM-Associated Signaling Pathways and Immunological Activities Are Identified Based on Treg Expression

**Figure 6:**
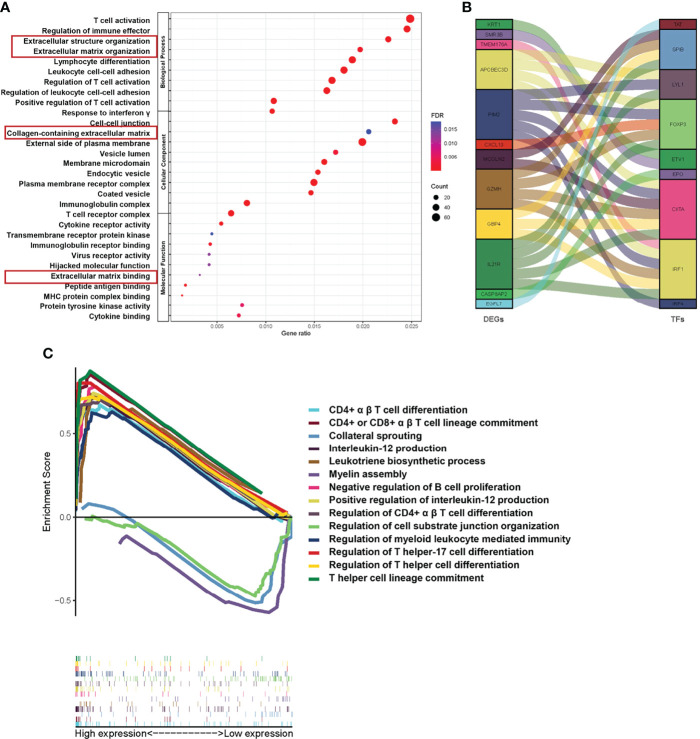
Functional pathway analysis based on Treg expression in TNBC. **(A)** The results of GO analyses between different Treg expression groups into three functional groups, including biological process (BP), cellular component (CC) and molecular function (MF). **(B)** Coexpression analysis of DEGs and immune-related transcription factors (TFs). **(C)** GSEA of significant Gene Ontology biological processes between the Treg-H and Treg-L groups based on the enrichment scores.

Since our data suggest that the risk model based on Treg expression levels stably predicts the prognosis of TNBC patients, we tried to select possible pathways between the Treg-H and Treg-L groups. To do this, GO and KEGG analyses were conducted based on the identified DEGs. In the GO analysis, DEGs were significantly enriched in immunological activity processes such as T-cell activation (GO: 0042110), regulation of immune effectors (GO: 0002697), and regulation of T-cell activation (GO: 0050863) (**Figure 6A**), while KEGG mainly identified immunological signaling pathways such as hematopoietic cell lineage (hsa04640), Th17-cell differentiation (hsa04659), and primary immunodeficiency (hsa05340) (**Figure S2**). Notably, ECM-associated processes, such as extracellular structure organization (GO: 0043062), collagen-containing extracellular matrix (GO: 0062023), and extracellular matrix binding (GO: 0050840), were also enriched according to GO analysis. Additionally, the coexpression of DEGs with transcription factors (TFs) (**Figure 6B**) and GSEA enrichment analysis (**Figure 6C**) showed similar immunological functions of the DEGs.

### Collagen Fiber Concentration and Orientation Distribution Are Associated With TNBC Progression

**Figure 7:**
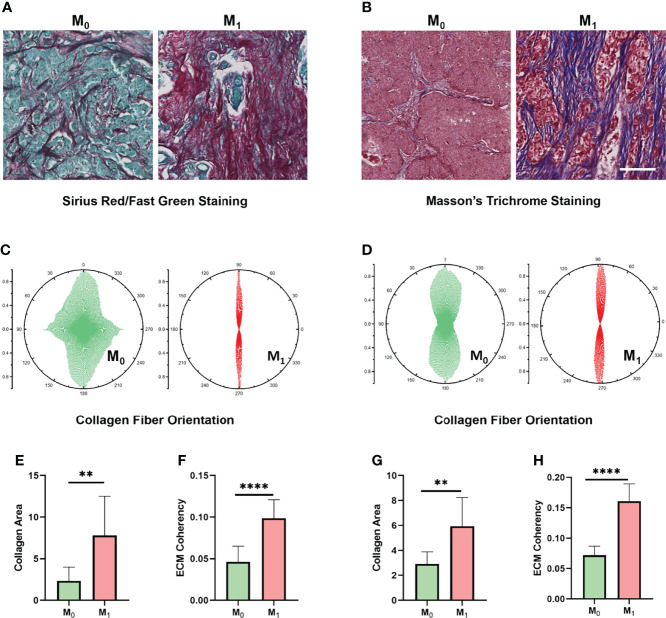
ECM characteristics are associated with TNBC metastasis in clinical samples. **(A)** Sirius red/Fast Green staining images of different metastatic statuses. **(B)** Masson’s trichrome staining images between different metastasis statuses. **(C)** Orientation distribution of collagen fibrils between different metastasis statuses based on Sirius red/Fast Green staining. **(D)** Orientation distribution of collagen fibrils between different metastasis statuses based on Masson’s trichrome staining. **(E)** Area of collagen fibrils between different metastasis statuses based on Sirius red/Fast Green staining. **(F)** Collagen fiber coherence between different metastasis statuses based on Sirius red/Fast Green staining. **(G)** Area of collagen fibrils between different metastasis statuses based on Masson’s trichrome staining. **(H)** Collagen fiber coherence between different metastasis statuses based on Masson’s trichrome staining. (scale bar, 100 μm). **p < 0.01, ****p < 0.0001.

Considering that our results indicate a critical role of the ECM-related pathway among the different Treg expression groups, we wondered whether ECM characteristics play a critical role in TNBC progression. To understand this, we performed Sirius red/Fast green staining and Masson staining on M_0_ (*n* = 10) and M_1_ (*n* = 10) TNBC samples to evaluate the characteristics of collagen fibrils that constitute the main component of ECM (**Figures 7A, B**). We compared the collagen area and organization of breast cancer patients in the M_0_ and M_1_ stages. Interestingly, the organization of collagen showed higher levels of orientation distribution in M_0_ TNBC patients (**Figures 7C, D**), suggesting a higher collagen fiber alignment (**Figures 7F, H**). In addition, the results showed an increased collagen concentration in the M_1_ TNBC tissues compared to the M_0_ TNBC tissues (**Figures 7E, G**). These results reveal an essential role of ECM characteristics in breast cancer metastasis.

### Collagen Fiber Concentration Within the 3D Matrix Regulates the Infiltration of Tregs

**Figure 8:**
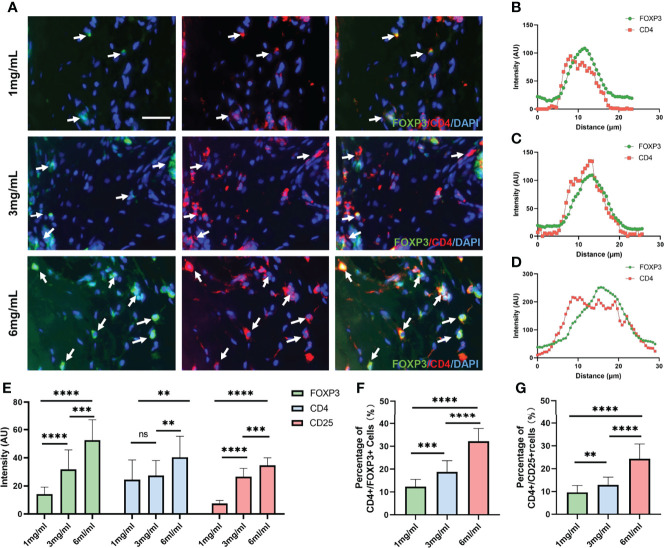
Collagen concentration regulates the expression levels of Treg biomarkers. **(A)** Immunofluorescence staining of FOXP3 and CD4 in Jurkat T cells encapsulated in different collagen concentrations. **(B)** Colocalization analysis of FOXP3 and CD4 in Jurkat T cells encapsulated in 1 mg/ml collagen matrix. **(C)** Colocalization analysis of FOXP3 and CD4 in Jurkat T cells encapsulated in 3 mg/ml collagen matrix. **(D)** Colocalization analysis of FOXP3 and CD4 in Jurkat T cells encapsulated in 6 mg/ml collagen matrix. **(E)** Average fluorescence intensity of FOXP3, CD4 and CD25 among different collagen concentration groups. **(F)** Percentage of CD4+/FOXP3+ T cells among different collagen concentration groups. **(G)** Percentage of CD4+/CD25+ T cells among different collagen concentration groups. (scale bar, 50 μm). ns, not significant. **p < 0.01, ***p < 0.001, ****p < 0.0001.

Given that ECM characteristics strongly correlate with TNBC metastasis, coupled with the previous evidence that ECM were essential for suppression of T-cell activity in the tumor microenvironment (18), we wondered whether ECM characteristics would affect the infiltration of Tregs and thus induce immune suppression. To evaluate the potential value of ECM, Jurkat T cells were capsulated in 3D high-, medium- and low-concentration collagen matrices (6 mg/ml vs. 3 mg/ml vs. 1 mg/ml). The expression levels of CD4, FOXP3 and CD25, specific biomarkers of Treg infiltration, were analyzed according to the average immunofluorescence intensity obtained in confocal images (**Figure 8A** and **Figure S3**). CD4 and FOXP3 were closely colocalized in each group (**Figures 8B–D**). In accordance with our prediction, the expression levels of FOXP3 and CD25 were significantly distinct among the different collagen concentration groups, which showed gradual increases with increasing collagen concentration (**Figure 8E**). Meanwhile, the expression level of CD4 remained similar in the 3 mg/ml and 1 mg/ml collagen concentration groups, while it was significantly higher in the 6 mgl/ml group (**Figure 8E**). We next quantified the cells that expressed both CD4 and FOXP3 to evaluate the infiltration of Tregs. Consistently, the results showed that with a higher collagen concentration, the percentage of CD4+/FOXP3+ cells were significantly increased (**Figure 8F**), and the percentage of CD4+/CD25+ cells were also significantly increased (**Figure 8G**). These observations suggest that increased collagen concentrations regulate Treg cell infiltration and thus may promote immune suppression in TNBC.

## Discussion

Due to the immunosuppressive ability in antitumor responses, a high abundance of tumor-infiltrating Tregs was associated with worse prognosis in malignant disease (9). TNBC is the most challenging molecular breast cancer because of metastatic capability and treatment resistance (2). Exploring the association between Tregs and TNBC will provide new insights for understanding TNBC immunosuppression and may pave the way for developing novel immune-based treatments. In this study, we first analyzed the association between Treg expression and the clinical characteristics of breast cancer based on a TCGA cohort. However, the following survival analysis is in contrast with the immunosuppressive role of Tregs. Interestingly, when we analyzed OS and DFS in each breast molecular subtype, our results showed a worse prognostic value of Treg expression levels in TNBC, which inspired us to establish a prognostic model based on Treg expression for further validation. Accordingly, both TCGA training and GEO validation cohorts support the accuracy and replicability of our prognostic model, suggesting the critical role of Tregs in TNBC. In addition, we evaluated the related signaling pathways enriched in different Treg expression groups to reveal the mechanisms that contribute to Treg regulation. Remarkably, ECM-associated signaling pathways were identified. Next, we established an *in vitro* 3D culture system based on the specific ECM variation between different TNBC stages, and we validated the potential role of collagen concentration in regulating the infiltration of Tregs.

Initially, CIBERSORT analysis was conducted to evaluate the abundance of Tregs, and our results exhibit significantly increased expression levels in cancer tissues compared to normal breast tissues, as well as higher expression in advanced clinical stages, which is in accordance with the role of Tregs in cancer progression (21–23). However, when we next analyzed the association between Treg expression and OS or DFS in breast cancer patients, the results showed a better OS in the Treg-H group, while DFS showed no significant difference, which is inconsistent with the above results and previous studies (22, 24). As reported previously, in some cancers, a high expression level of Tregs often correlates with a favorable prognosis, especially colorectal cancer (CRC) (25). Considering the high heterogeneity of breast cancer, we then explored the prognostic value of Tregs in each breast cancer subtype separately and found a favorable prognosis of Tregs in the luminal subtype in contrast to a poor prognostic value in TNBC. This is probably due to the distinct TME status between the two molecular subtypes. In addition, the regulators of Tregs, including PD-1, PD-L1, and CTLA-4, also showed various expression levels among the two molecular subtypes (26).

Consistent with previous demonstrations (8, 10), the survival analysis in TNBC suggests that high Treg expression indicates worse prognosis. We subsequently identified DEGs among the Treg-H and Treg-L groups, and the expression levels of immune-related genes and specific biomarkers of Tregs, such as CD4, FOXP3, and CD25, showed significant differences between the two groups, supporting the distinct TME between the Treg-H and Treg-L groups. Among the 12 core genes identified in the Treg-related prognostic model, most genes hold critical roles in immunity activity. ARS-interacting multifunctional protein 1 (AIMP1) induces the production of inflammatory cytokines from immune cells and affects the antitumor response by promoting TH1 polarization (27). In addition, dimethylarginine dimethylaminohydrolase 1 (DDAH1) is implicated in the establishment of VM by TNBC cells, which is strongly associated with a worse prognosis in TNBC (28), suggesting a collaborative role of VM in immunosuppression (29). Importantly, as reported previously, TILs in breast cancer can form organized tertiary lymphoid structures (TLSs) characterized by CXCL13+ T follicular helper (Tfh) cells (30), and high levels of CXCL13+ T cells are associated with the response to PD-L1 blockade in TNBC according to a single-cell RNA- and ATAC-sequencing analysis in 22 patients with advanced TNBC treated with paclitaxel alone or in combination with the anti-PD-L1 atezolizumab (31). Moreover, Egfl7, also known as VE-statin, promotes cancer suppression by inhibiting TIL-associated endothelial molecules (32). This evidence supports the effectiveness and reliability of our prognostic model based on Treg expression.

Besides immunological signaling pathways, ECM-associated activities were enriched among the different Treg expression groups. Consistent with previous evidence, we found that increasing collagen concentration and alignment of collagen fibers are associated with TNBC progression (33–36). It has been demonstrated that treatment targeting collagen crosslinking improves T-cell migration and anti-PD-1 treatment, suggesting the barrier role of collagen in the cancer immune response (37). In addition, collagen density can also regulate the activity of tumor-infiltrating CD8+ T cells (18). Interestingly, we identified the potential role of collagen fibers in regulating Treg cell infiltration through functional analysis. More importantly, our *in vitro* 3D experimental validation shows that increasing collagen concentration promotes the expression of CD4 and FOXP3 and the percentage of CD4+/FOXP3+ T cells, which suggests the regulatory role of collagen concentration in immunosuppression.

Due to the direct 3D experimental evidence that collagen fiber concentration regulates Treg cell infiltration, combined with our database analysis, we observed the immunosuppressive role of collagen fiber concentration, suggesting the potential treatment value of targeting ECM and the prognostic genes included in our risk mode during TNBC progression. Presently, treatment targeting matrix metalloproteinases (MMPs) and lysyl oxidase (LOX) to reverse ECM stiffness shows promising antitumor effects (37, 38). All this evidence reveals that intervention of the ECM-related pathway might be a promising strategy for improving the efficacy of anticancer responses.

## Conclusion

In summary, our comprehensive RNA-seq analysis of the TCGA database supports the association between Treg expression and breast cancer progression. Moreover, a high expression level of Tregs predicts a worse prognosis in the TNBC subtype. A predictive model based on Treg expression may accurately predict the outcome of TNBC patients. Moreover, increasing collagen density may promote Treg cell infiltration and thus induce an immunosuppressed TME, which could be a promising therapeutic target to improve TNBC outcomes.

## Data Availability Statement

The datasets presented in this study can be found in online repositories. The names of the repository/repositories and accession number(s) can be found in the article/**Supplementary Material**.

## Ethics Statement

The studies involving human participants were reviewed and approved by institutional Review Board of the First Affiliated Hospital of Xi’an Jiaotong University. The patients/participants provided their written informed consent to participate in this study. The animal study was reviewed and approved by Institutional Animal Care Committee of Xi’an Jiaotong University. Written informed consent was obtained from the individual(s) for the publication of any potentially identifiable images or data included in this article.

## Author Contributions

HG and QT contributed to the study design and performed the experiments. YNJ contributed to the pathological analysis of ECM. YZ, LZ, YL, and YM contributed to data collection. HG, QT, and BW performed statistical analysis and interpretation. HG and BW drafted the manuscript. All authors contributed to critical revision of the final manuscript.

## Funding

This study was supported by the National Natural Science Foundation of China (No.82173277) and International Cooperation Foundation Projects of Shannxi Province (No. 2019KW-033).

## Conflict of Interest

The authors declare that the research was conducted in the absence of any commercial or financial relationships that could be construed as a potential conflict of interest.

## Publisher’s Note

All claims expressed in this article are solely those of the authors and do not necessarily represent those of their affiliated organizations, or those of the publisher, the editors and the reviewers. Any product that may be evaluated in this article, or claim that may be made by its manufacturer, is not guaranteed or endorsed by the publisher.

## Supplementary Material

Supplementary Figure 1 Abundance of tumor-infiltrating immune cells analyzed by the CIBERSORT algorithm **(A)**. Abundance of tumor-infiltrating immune cells based on an RNA-seq analysis of breast cancer patients in the TCGA database. **(B)** Heatmap of the specific immune cell infiltration between normal and breast cancer tissues. **(C)** Differences in immune cell infiltration between normal and breast cancer tissues.

Supplementary Figure 2 Results of KEGG analyses between different Treg expression groups.

Supplementary Figure 3 Immunofluorescence staining of CD4 and CD25 in Jurkat T cells encapsulated in different collagen concentrations.
