# Supplementary figures and images for: 3D Collagen Fiber Concentration Regulates Treg Cell Infiltration in Triple Negative Breast Cancer
Source: Front Immunol. 2022 Jun 14;13:904418. doi: 10.3389/fimmu.2022.904418 (PMC9237245; doi:10.3389/fimmu.2022.904418)

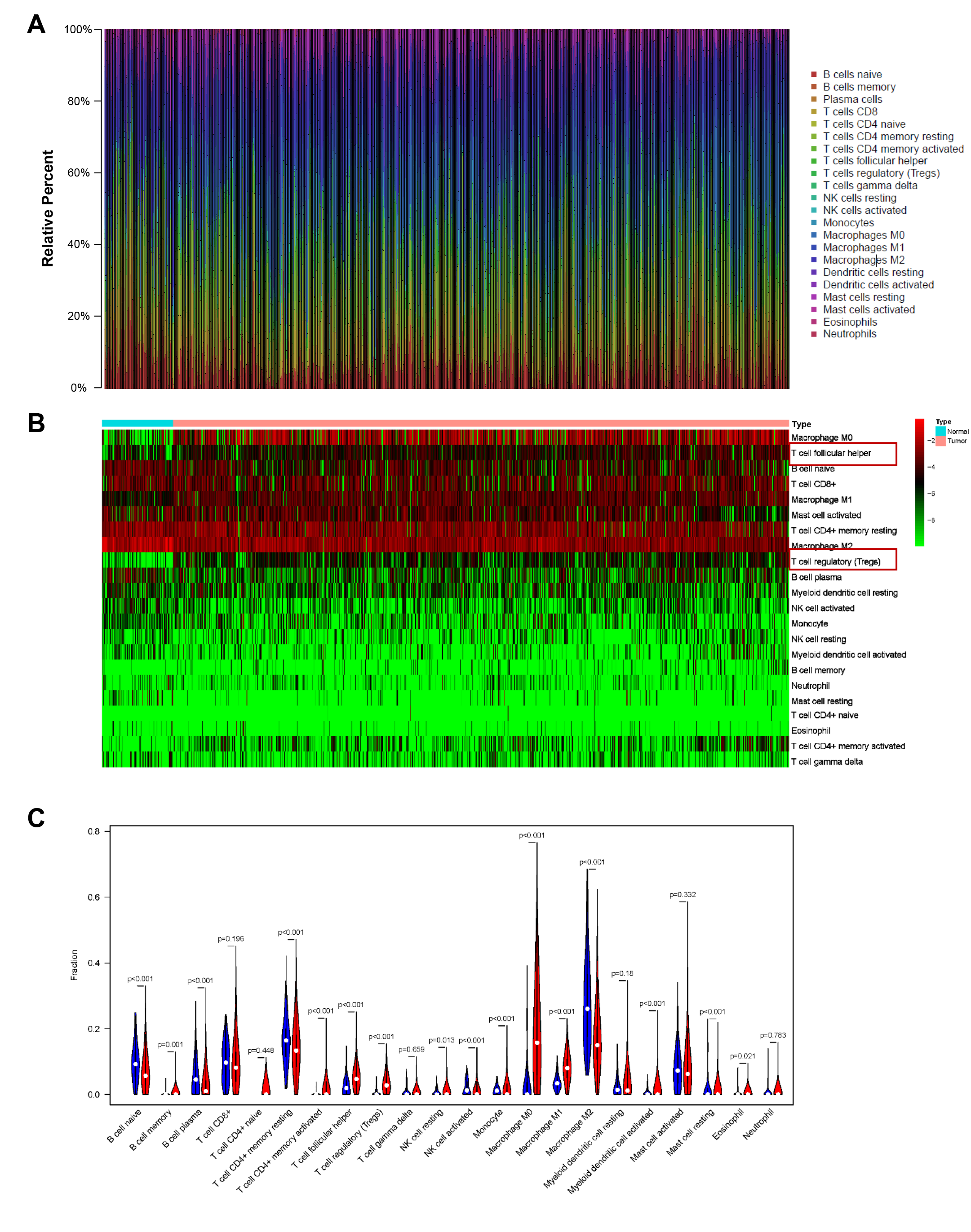

Supplement: Supplementary Figure 1 — Abundance of tumor-infiltrating immune cells analyzed by the CIBERSORT algorithm (A). Abundance of tumor-infiltrating immune cells based on an RNA-seq analysis of breast cancer patients in the TCGA database. (B) Heatmap of the specific immune cell infiltration between normal and breast cancer tissues. (C) Differences in immune cell infiltration between normal and breast cancer tissues. [file Image_1.tif]

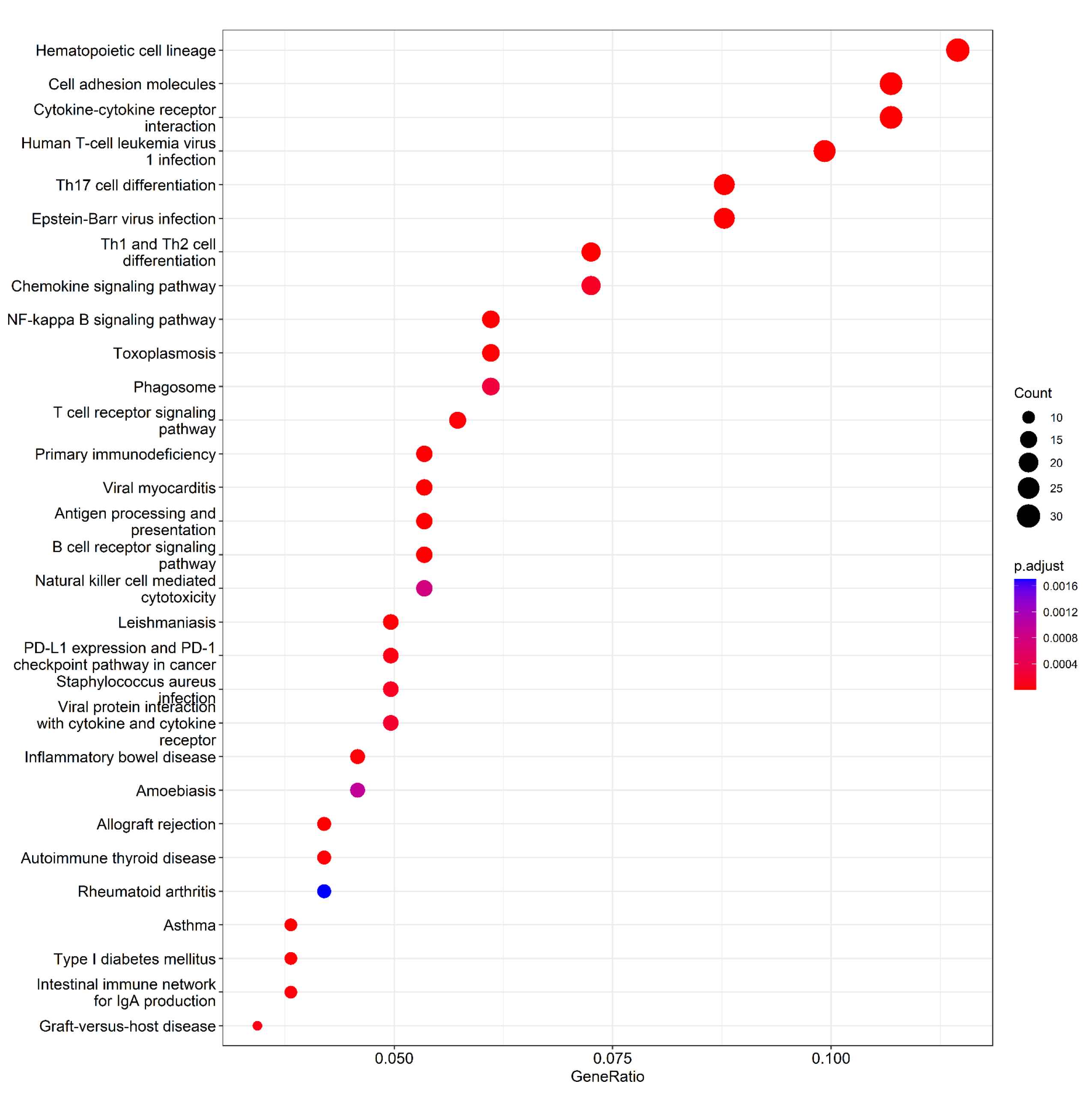

Supplement: Supplementary Figure 2 — Results of KEGG analyses between different Treg expression groups. [file Image_2.tif]

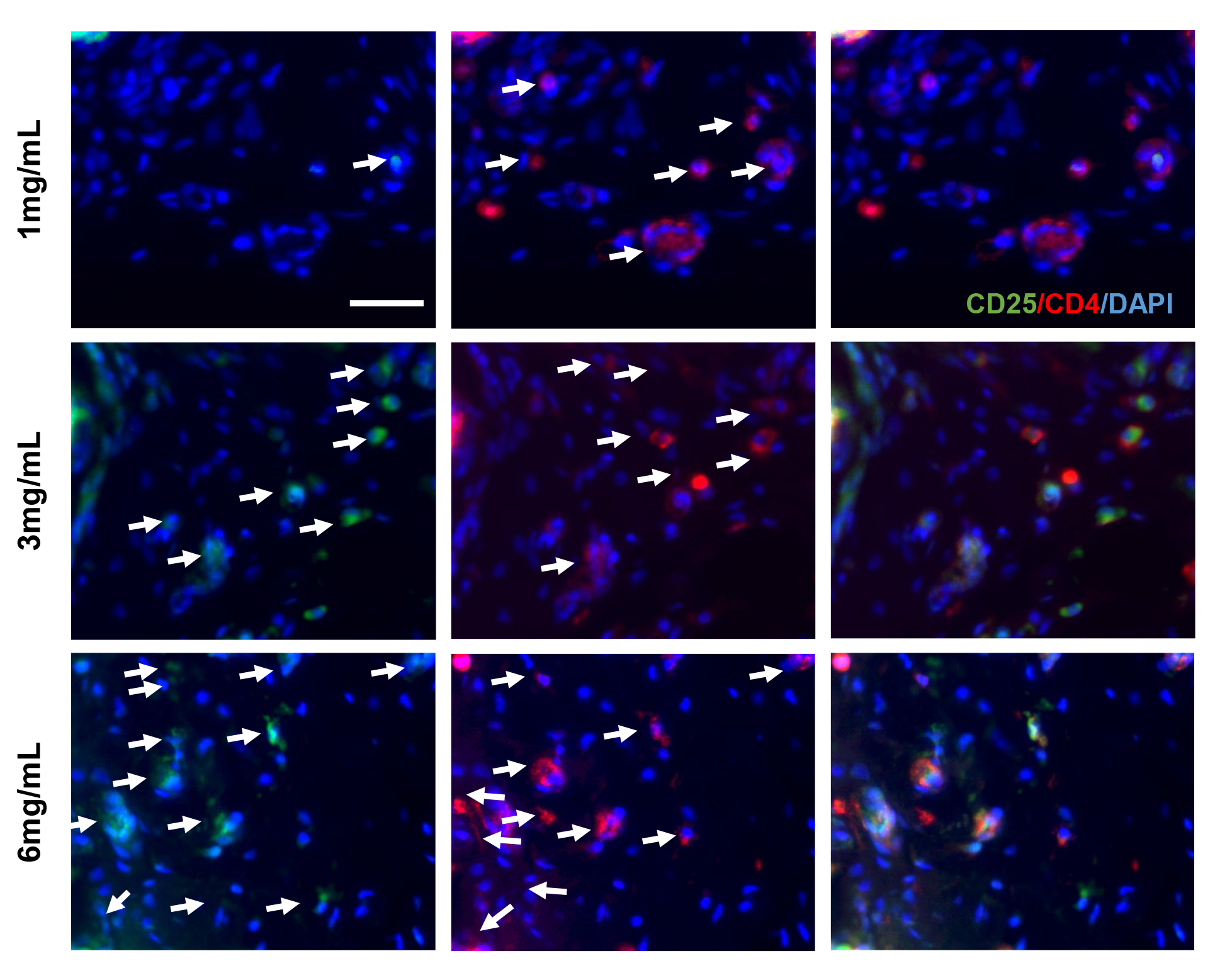

Supplement: Supplementary Figure 3 — Immunofluorescence staining of CD4 and CD25 in Jurkat T cells encapsulated in different collagen concentrations. [file Image_3.tif]
